# Impact of B-type natriuretic peptide level on the risk of left atrial appendage thrombus in patients with nonvalvular atrial fibrillation: a prospective study

**DOI:** 10.1186/s12947-016-0047-6

**Published:** 2016-01-16

**Authors:** Rojina Pant, Mita Patel, Enrique Garcia-Sayan, Marwan Wassouf, Oliver D’Silva, Richard F. Kehoe, Rami Doukky

**Affiliations:** 1 Division of Cardiology, Advocate Illinois Masonic Medical Center, Chicago, IL USA; 2 Division of Cardiology, Rush University Medical Center, Chicago, IL USA; 3 Division of Cardiology, Medical College of Wisconsin, Milwaukee, WI USA; 4 Division of Cardiology, Mount Sinai Hospital, Chicago, IL USA; 5 Department of Medicine, Fairview Hospital, Cleveland, OH USA; 6 Division of Adult Cardiology, John H. Stroger, Jr. Hospital of Cook County, 1901 W. Harrison St, Chicago, IL 60612 USA

**Keywords:** Atrial fibrillation, B-type natriuretic peptide, Left atrial appendage thrombus, Spontaneous echo contrast

## Abstract

**Background:**

The impact of B-type natriuretic peptide (BNP) level on the risk of left atrial appendage (LAA) thrombus in patients with nonvalvular atrial fibrillation (NVAF) has not been prospectively studied.

**Methods:**

In two academic medical centers, we obtained BNP levels immediately prior to transesophageal echocardiogram performed to exclude LAA thrombus in patients with NVAF.

**Results:**

Among 261 subjects (mean age 65 ± 12 years; 30 % women) with NVAF, 17 (6.5 %) had LAA thrombus and 85 (32.6 %) had at least mild spontaneous echo contrast (SEC). Mean BNP level was significantly higher in patients with LAA thrombus [775 ± 678 vs. 384 ± 537, *P* = 0.001]. Receiver operator characteristics analysis demonstrated that BNP has a good discriminatory capacity for LAA thrombus (area under the curve, 0.74; 95 % confidence interval [CI], 0.63–0.85; *P* = 0.001); BNP ≥ 67 pg/mL was 100 % sensitive and 20 % specific for LAA thrombus. Multivariate logistic regression analysis demonstrated that BNP was not independently associated with LAA thrombus (odds-ratio, 1.05 per 100 pg/mL increment; CI, 0.99–1.12; *P* = 0.127) after adjusting for CHA_2_DS_2_-VASc score; while the latter was independently associated with LAA thrombus after adjusting for BNP level (odds-ratio, 1.46 per CHA_2_DS_2_-VASc point; CI, 1.09–1.96; *P* = 0.011). Nonetheless, BNP was associated with SEC in univariate and multivariate analysis, after adjusting for the CHA_2_DS_2_-VASc score, (odds-ratio, 1.08; CI, 1.02–1.14; *P* = 0.005).

**Conclusions:**

BNP is predictive of SEC. However, it does not provide significant incremental value in the prediction of LAA thrombus.

## Background

Systemic thromboembolism is a serious complication in patients with nonvalvular atrial fibrillation (NVAF) [1]. Over the past several years, various types of scoring systems, such as the CHADS_2_ and CHA_2_DS_2_-VASc scores have been developed for predicting the risk of embolic events in NVAF patients [2, 3]. These clinical risk scores seem to offer a modest discriminatory value for the individual patients [2]. Biomarkers of myocardial stress such as B-type natriuretic peptide (BNP) have a large body of evidence showing association with clinical events and may help refine risk assessment in NVAF patients [4]. Atrial dysfunction is an established risk factor for thrombus formation in NVAF patients and BNP has been shown to originate from dysfunctional and stressed atrial myocadium in these patients [5–7]. Moreover, elevated left ventricular filling pressure leads to left atrial blood stasis and thrombus formation, which respectively manifest on transesophageal echocardiography (TEE) as spontaneous echo contrast (SEC) and left atrial appendage (LAA) thrombus [8–10]. Thus, BNP, a surrogate for left ventricular filling pressure, may help identify patients at risk for left atrial blood stasis and LAA thrombus [11, 12]. Indeed, in retrospective non-concomitant assessment of BNP levels and LAA thrombus by TEE, our group has demonstrated an association between plasma BNP level and the formation of SEC and LAA thrombi in patients with NVAF, independent of clinical covariates [13]. The present study aims to prospectively and concomitantly investigate the predictive value of BNP level in identifying LAA thrombus and SEC formation in patients with NVAF.

## Methods

### Patient population and study design

We prospectively enrolled consecutive adult patients with NVAF who were referred to undergo TEE to “rule-out” LAA thrombus at the echocardiography laboratories of Rush University Medical Center (Chicago, IL) and Advocate Illinois Masonic Medical Center (Chicago, IL) in the period between January 7, 2011 and November 14, 2013. Eligible patients underwent plasma BNP level measurement from a blood sample drawn immediately before the reference TEE.

Patients with valvular atrial fibrillation were excluded; those were patients with mitral stenosis, mitral regurgitation greater than 2+ in severity (scale, 0–4), or any mitral valve surgical or percutaneous intervention. Additionally, patients with complex congenital heart disease and orthotopic heart transplantation were excluded. Patients with isolated aortic valvular disease, aortic valve prosthesis, and right-sided valvular heart disease were not excluded. All subjects signed an informed and HIPAA consent prior to enrollement. The study was funded by an internal research grant and approved by the institutional review board of each participating institution.

### Clinical data

Data including patient demographics, comorbidities, anticoagulation status, and antiplatelet use were prospectively collected prior to the TEE. Atrial fibrillation chronicity was classified as: 1) paroxysmal, defined as spontaneously reverting to sinus rhythm within 7 days; 2) persistent, defined as lasting more than 7 days but less than 12 months, or any atrial fibrillation events terminated by electrical or chemical cardioversion or radiofrequency ablation within 12 months of onset; or 3) permanent, defined as events lasting more than 12 months [14]. The CHADS_2_ score was calculated from the sum of risk predictors of congestive heart failure, hypertension, age ≥75 years, diabetes mellitus, stroke or transient ischemic attack; weighing each risk predictor by “1” except for prior stroke/transient ischemic attack which was weighed by “2” [2]. The CHA_2_DS_2_-VASc score was calculated from the sum of the risk factors of congestive heart failure, hypertension, age 65–74 or ≥75 years, diabetes mellitus, stroke/transient ischemic attack, vascular disease, female gender; weighing each by “1” except for stroke/transient ischemic attack and age ≥75 years which were weighed by “2” [3, 15]. Congestive heart failure was defined as a history of clinical heart failure or left ventricular ejection fraction (LVEF) < 40 %. Vascular disease was defined as peripheral arterial disease, complex aortic plaque, or prior myocardial infarction [3]. The left ventricular end-systolic and end-diastolic volumes and LVEF were prospectively determined using the biplane Simpson’s method [16] from a transthoracic echocardiogram acquired immediately prior to the reference TEE [16]. The LA volume was measured using single-plane Simpson’s method in the apical 4 chamber view [16]. The left ventricular mass was calculated using the Devereux formula [17]. All volume and mass measurements were indexed to the body surface area.

### B-type natriuretic peptide

A blood sample for BNP level was drawn immediately prior to induction of sedation for the reference TEE. Blood samples were processed at the respective inpatient laboratory of the participating institutions. BNP level was expressed in pg/mL.

### Transesophageal echocardiography

**Fig. 1:**
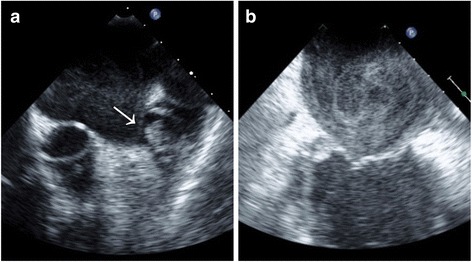
Representative Examples. a, left atrial appendage thrombus (arrow); b, left atrial spontaneous echo contrast. Reproduced from Doukky et al. [29], with permission

TEE studies were performed under moderate or deep sedation, as clinically appropriate. An expert National Board of Echocardiography certified echocardiographer, who was blinded to BNP and clinical data, reviewed all TEE images to determine the presence or absence of LAA thrombus, defined as a circumscribed and uniformly echo-dense intracavitary mass distinct from the underlying left atrial or LAA endocardium and the pectinate muscles, and present in more than one imaging plane (Fig. 1a) [18]. LAA sludge, defined as a dynamic gelatinous, precipitous echodensity, without a discrete mass, present throughout the cardiac cycle, was categorized as LAA thrombus [19, 20]. SEC was defined as dynamic “smoke-like” echoes with the characteristic swirling motion with optimal gain setting during the entire cardiac cycle (Fig. 1b) and was classified from 1 to 4 based on the criteria described by Fatkin et al [21]. Peak LAA emptying velocity at the LAA orifice was measured using pulsed wave Doppler and averaged over 3–5 beats.

### Outcomes

The primary outcome of the study was TEE-identified LAA thrombus. The secondary outcome was TEE-identified SEC.

### Statistical analysis

The two-tailed Student’s *t*-test was used to compare normally distributed continuous variables, which were expressed as mean ± standard deviation. The Mann–Whitney test was used to compare skewed data. The chi-square test was used to compare categorical variables, which were expressed as frequencies and percentages. The Spearman’s method was used to evaluate linear correlations.

The receiver operating characteristic (ROC) methodology was used to analyze the discriminatory capacity of BNP and CHA_2_DS_2_-VASc score in predicting LAA thrombus. ROC analyses were expressed as curve plots and calculated area under the curve (AUC) with 95 % confidence intervals (CI) and associated *P* value representing the likelihood of the null hypothesis (AUC = 0.5).

The association between BNP level (in 100 pg/mL increments) as main independent variable, and LAA thrombus or SEC (outcome variable) was analyzed in univariate and multivariate binary logistic regression models. In multivariate binary logistic regression models, we initially adjusted for the CHA_2_DS_2_-VASc score only, and in separate models we adjusted for the CHA_2_DS_2_-VASc score, LVEF, and oral anticoagulant use. Due to significant collinearity between BNP level and LA volume index (Spearman’s *r* = 0.41; *P* < 0.001), LA volume index was not included in multivariate models. Risk was expressed as odds-ratios (OR) with CI. Goodness-of-fit for multivariate models was confirmed using Hosmer and Lemeshow test.

All tests were 2-tailed, and a *P* value <0.05 was considered statistically significant. PASW-22 software (IBM, Inc., Armonk, NY) was used for all data analyses.

## Results

A total of 261 patients (mean age, 65 ± 12 years; 30 % women) with NVAF were prospectively enrolled to undergo plasma BNP level measured in a blood sample drawn immediately prior to their clinically indicated TEE; 199 (76 %) were recruited from Rush University Medical Center and 62 (24 %) from Advocate Illinois Masonic Medical Center. The leading indications for TEE were “ruling-out” LAA thrombus prior to direct current cardioversion (39 %), catheter ablation (46 %), or implantation or testing of implantable cardioverter-defibrillator (10 %). At the time of the TEE, 34 (13 %) subjects were in sinus rhythm; the rest were in atrial dysrhythmia. Among the study subjects, 17 (6.5 %) had a confirmed LAA thrombus by TEE. Table 1 compares the baseline characteristics of patients with and without LAA thrombus. Notably, patients with LAA thrombus had a significantly higher mean CHA_2_DS_2_-VASc and CHADS_2_ scores (both *P* values = 0.001), lower mean LVEF, larger LA volume index, lower mean LAA emptying velocity, higher prevalence of heart failure, and had a trend towards higher creatinine levels. Patients with LAA thrombus had statistically insignificant higher rates of Warfarin usage. Chronicity of NVAF was not different between patients with versus without LAA thrombus (Table 1).

**Table 1:** Baseline Characteristics

		LAA Thrombus		
	All Subjects	Present	Absent	*P* value
	*N* = 261	*N* = 17	*N* = 244	
Age (years)	65 ± 12	69 ± 10	65 ± 12	0.190
Age ≥ 75 years	56 (21)	5 (29)	51 (21)	0.409
Age ≥ 65 years	150 (58)	13 (76)	137 (56)	0.101
Female	79 (30)	5 (29)	74 (30)	0.937
Hypertension	196 (75)	15 (88)	181 (74)	0.195
Diabetes mellitus	67 (26)	7 (41)	60 (25)	0.130
Congestive heart failure	102 (39)	13 (76)	89 (36)	0.001
History of stroke or TIA	35 (13)	5 (29)	30 (12)	0.045
Vascular disease	53 (20)	5 (29)	48 (20)	0.334
Dyslipidemia	126 (48)	9 (53)	117 (48)	0.691
BMI (Kg/m^2^)	31.4 ± 8.5	28 ± 5.7	31.7 ± 8.7	0.091
Creatinine (mg/dL)	1.3 ± 1.1	1.9 ± 1.9	1.2 ± 1.0	0.076*
Warfarin	124 (48)	10 (59)	114 (47)	0.334
NOACs	48 (18)	3 (18)	45 (18)	0.935
Antiplatelet	140 (54)	9 (53)	131 (54)	0.952
CHADS_2_ score	1.9 ± 1.3	2.9 ± 1.3	1.8 ± 1.3	0.001*
CHA_2_DS_2_-VASc Score	3.0 ± 1.8	4.4 ± 1.6	3.0 ± 1.8	0.001*
AF chronicity				0.789
Paroxysmal	67 (26)	5 (29)	62 (25)	
Persistent	107 (41)	7 (41)	100 (41)	
Permanent	34 (13)	3 (18)	31 (13)	
Unknown	53 (20)	2 (12)	51 (21)	
LVEF (%)	47 ± 17	34 ± 13	48 ± 17	0.002
LVEDV index (mL/m^2^)	54 ± 29	69 ± 34	53 ± 29	0.054
LV mass index (g/m^2^)	103 ± 41	113 ± 29	102 ± 41	0.311
LA volume index (mL/m^2^)	33 ± 16	42 ± 18	33 ± 16	0.035
Degree of SEC (scale 0–4)	0.6 ± 1.1	3.0 ± 1.1	0.4 ± 0.9	<0.001*
SEC present	85 (33)	17 (100)	68 (28)	<0.001
LAA emptying velocity (cm/s)	42 ± 22	25 ± 11	44 ± 22	0.001
BNP (pg/mL)	409 ± 554	775 ± 678	384 ± 537	0.001*

Data presented as mean ± standard deviation or number (%)

*T*IA transient ischemic attack, *BMI* body mass Index, *NOACs* novel oral anticoagulants, *AF* atrial fibrillation, *LVEF* Left ventricular ejection fraction, *LVEDV* left ventricular end-diastolic volume, *LV* left ventricular, *LA* left atrial, *SEC* spontaneous echo contrast, *LAA* left atrial appendage, *BNP* B-type natriuretic peptide, *Mann–Whitney test

### BNP and LAA Thrombus

**Fig. 2:**
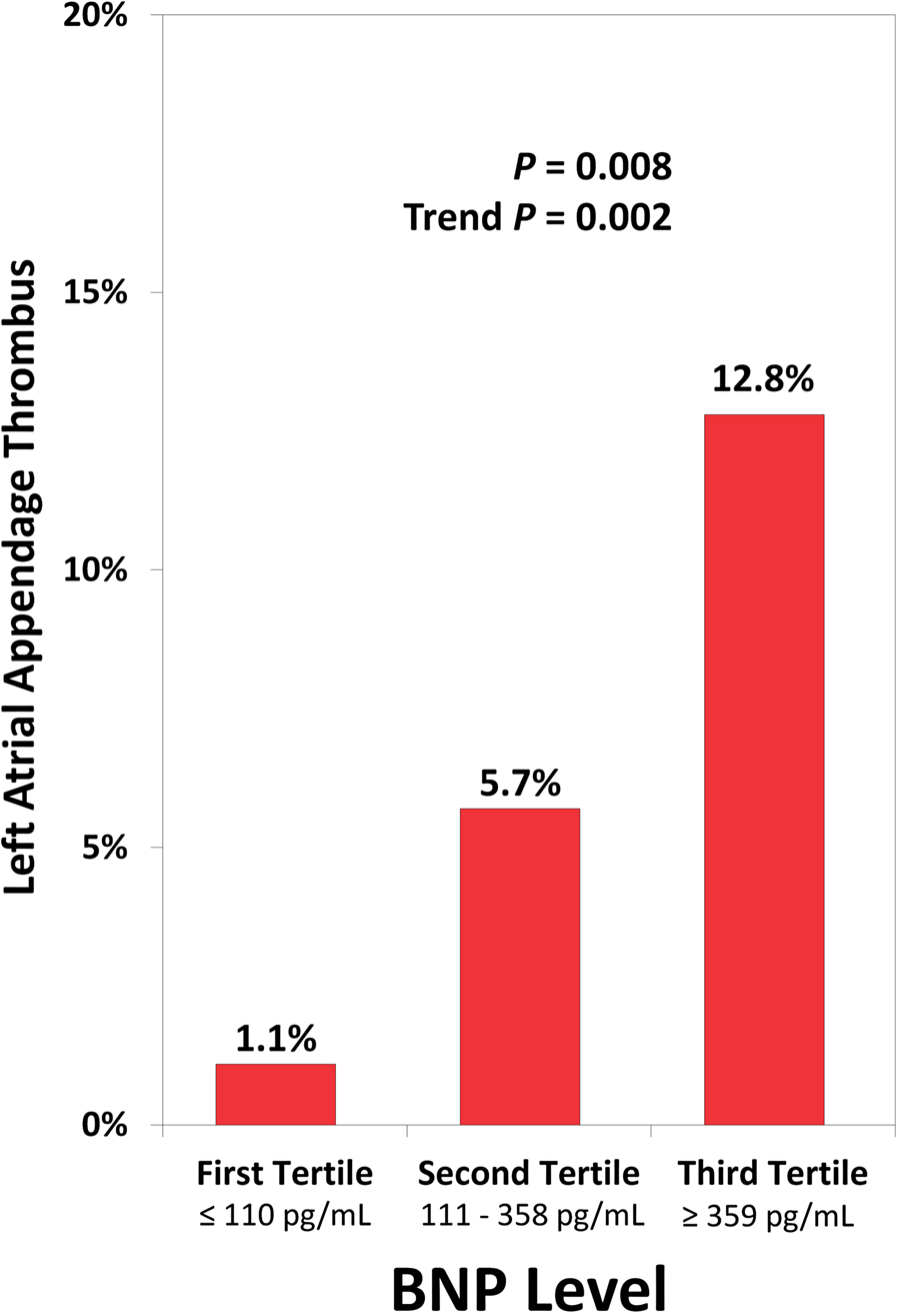
Prevalence of Left Atrial Appendage Thrombus according to BNP level

**Fig. 3:**
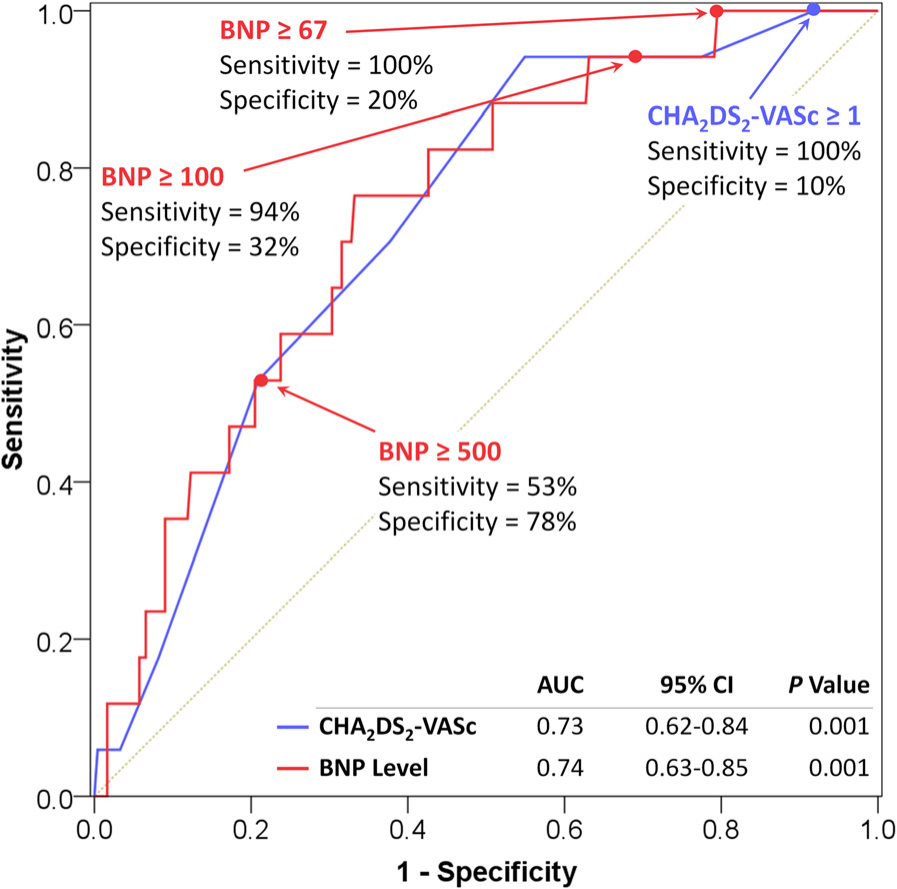
Receiver Operating Characteristic Curves in Diagnosing LAA Thrombus. LAA, left atrial appendage; AUC, area under the curve; CI, confidence intervals

Subjects with LAA thrombus had a significantly higher mean BNP level than those without LAA thrombus [775 ± 678 vs. 384 ± 537, *P* = 0.001] (Table 1). Furthermore, there was a significant stepwise increase (P for trend = 0.002) in the prevalence of LAA thrombus with increasing BNP level (Fig. 2). As shown in Fig. 3, ROC analysis demonstrated that BNP had a good discriminatory capacity for LAA thrombus (AUC, 0.74; CI, 0.63–0.85; *P* = 0.001). BNP level ≥ 67 pg/mL was 100 % sensitive and 20 % specific for LAA thrombus, whereas BNP ≥ 100 was 94 % sensitive and 32 % specific and BNP ≥ 500 was 53 % sensitive and 78 % specific for LAA thrombus. CHA_2_DS_2_-VASc score had similar discriminative capacity (AUC, 0.73; CI, 0.62–0.84; *P* < 0.001); a score of ≥1 was 100 % sensitive for LAA thrombus (Fig. 3).

BNP was associated with LAA thrombus in univariate analysis (OR, 1.08 per 100 pg/mL increment; CI, 1.02–1.15; *P* = 0.010). However, in multivariate logistic regression analysis, adjusting for the CHA_2_DS_2_-VASc score, BNP was not independently associated with LAA thrombus (OR, 1.05 per 100 pg/mL increment; CI, 0.99–1.12; *P* = 0.127), while the CHA_2_DS_2_-VASc score was independently associated with LAA thrombus after adjusting for BNP (OR, 1.46 per point; CI, 1.09–1.96; *P* = 0.011). In the latter model, BNP level did not seem to provide significant incremental predictive value (Δχ^2^ = 2.08, *P* = 0.149) to the CHA_2_DS_2_-VASc score. In an expanded (over-fitted) multivariate logistic regression analysis model, adjusting for CHA_2_DS_2_-VASc score, LVEF, and oral anticoagulants use, BNP was not independently predictive of LAA thrombus (OR, 1.04 per 100 pg/mL increment; CI, 0.97–1.12; *P* = 0.229).

### BNP and spontaneous echo contrast

Out of 261 study subjects, 85 (33 %) had at least mild SEC, including all 17 (100 %) individuals with LAA thrombus. The CHA_2_DS_2_-VASc score was associated with SEC (OR, 1.44 per 1 point increment; CI, 1.23–1.68; *P* < 0.001). There were modest but highly significant correlations between the degree of SEC (scale, 0–4) and both the CHA_2_DS_2_-VASc score (Spearman *r* = 0.29, *P* < 0.001) and BNP (Spearman *r* = 0.32, *P* < 0.001). In a multivariate logistic regression analysis model that included BNP level and the CHA_2_DS_2_-VASc score, BNP was independently predictive of SEC (OR, 1.08 per 100 pg/mL increment; CI, 1.02–1.14; *P* = 0.005). In this model, the CHA_2_DS_2_-VASc score was also predictive of SEC (OR, 1.35; CI, 1.15–1.59; *P* < 0.001). In an expanded logistic regression model, that included BNP level, CHA_2_DS_2_-VASc score, LVEF, and oral anticoagulant use, BNP was independently predictive of SEC (OR, 1.09; CI, 1.02–1.15; *P* = 0.007).

## Discussion

This is the first study to prospectively and concomitantly correlate the BNP level with LAA thrombus and SEC in patients with NVAF. This study showed that plasma BNP level was significantly higher in patients with LAA thrombus than in those without LAA thrombus. Despite small number of LAA thrombus events, we observed a stepwise relationship between BNP level (in tertiles) and risk of LAA thrombus. Thus, BNP level may help in identifying patients at risk for LAA thrombus. BNP level was independently predictive of SEC but not of LAA thrombus.

BNP is a neurohormone secreted from the myocytes mainly in response to increased wall tension such as volume or pressure overload and has been shown to be elevated in patients with NVAF [22]. Moreover, BNP has also been shown to predict future AF [23, 24] and risk of thromboembolism [13, 25]. Recent studies have shown BNP to be a valuable marker for general cardiovascular risk stratification in patients with NVAF [26]. Pathophysiologically, increased LV filling pressure causes atrial stretch, which is a well-known reason for development of NVAF [27]. Loss of effective atrial contraction during atrial fibrillation coupled with elevation in the left ventricular filling pressure can lead to left atrial blood stasis, manifesting as SEC, and subsequent risk for LAA thrombus [28, 29]. BNP level correlates with left ventricular filling pressure (6); thus, it may aid in predicting SEC and LAA thrombus. Indeed, in a retrospective analysis of 136 patients with NVAF our group demonstrated an association between BNP, a surrogate of LV filling pressure, and LAA thrombus and SEC [13]. The study demonstrated that BNP was predictive of LAA thrombus independent of the CHADS_2_ and CHA_2_DS_2_-VASc scores, which are known predictors of LAA thrombus and systemic embolization in NVAF. Additionally, the study showed that all patients with LAA thrombus had BNP level > 500 pg/mL [13]. Moreover, in another recent retrospective study of 524 non-anticoagulated NVAF patients, Ochiumi et al., [25] showed that BNP was an independent predictor of LAA thrombus, and identified plasma BNP level of 251 pg/mL as a useful cutoff for predicting LAA thrombus. Notably, both of the aforementioned studies were limited by retrospective design, incidental rather than systematic BNP level assessment with TEE, and non-concomitant BNP measurement with TEE.

The present investigation partially validates the finding of previous retrospective studies, [13, 25] as BNP was predictive of LAA thrombus only in univariate analysis but not in multivariate analysis after adjusting for the CHA_2_DS_2_-VASc score. However, BNP was predictive of SEC in univariate and multivariate analyses. Since SEC is a surrogate for LAA thrombus formation and thromboembolic events, [30] it is likely that lack of independent association between BNP and LAA thrombus is due to small sample size and limited number of events (type II error). Moreover, the present study demonstrated that LAA thrombus was present at much lower BNP level (67 pg/mL) than previously shown [13, 25]. This significant difference in the BNP level associated with LAA thrombus is likely due to the fact that, given our prospective design, BNP levels were collected immediately prior to the TEE exam, when patients may have been near euvolemic state; whereas in other studies BNP was obtained when clinically indicated, at the height of patients’ symptoms. Moreover, all patients in the present study had a BNP level measured. This was not the case in other published studies in which BNP levels were incidentally available among patients who underwent TEE studies, thus introducing a clear selection bias. Therefore, the current work is unique in several important aspects: 1) this is the only prospective study to investigate the impact of BNP on the risk of LAA thrombus; 2) the study was designed to include dual-center data; 3) concomitant BNP and TEE assessment; 4) we investigated consecutively enrolled patients to undergo BNP level measurement prior to TEE, which eliminates the bias introduced by analyzing patients who happen to have incidental BNP measurement.

The association between BNP and SEC suggests that elevated left ventricular filling pressure is associated with left atrial blood stasis manifesting as SEC. This association was independent of other factors such as LVEF, and oral anticoagulants. This finding also support the findings regarding LAA thrombus since SEC is the precursor for LAA thrombus formation.

It was notable that 13 patients were found to have LAA thrombus despite oral anticoagulant use. This observation likely reflects the recognition of elevated thromboembolic risk by the treating physician. It also indicates that oral anticoagulants are not fully protective of LAA thrombus among high risk NVAF patients.

The present investigation has two important clinical implications: 1) BNP level can be used as a useful predictor for LAA thrombus and SEC; 2) Patients with very low BNP levels seem to be at low risk for LAA thrombus and perhaps systemic embolic events.

The study is limited by a relatively small sample size and infrequent LAA thrombus events, which impaired our ability to analyze many covariates within a given multivariate logistic regression model. However, the secondary SEC analyses were consistent with the primary endpoint analyses; thus adding validity to the study conclusions.

## Conclusions

In patients with NVAF, BNP is predictive of SEC independent of clinical risk factors encompassed in the CHA_2_DS_2_-VASc score. BNP level can help predicting LAA thrombus in patients with NVAF, but does not seem to provide significant incremental predictive value beyond the CHA_2_DS_2_-VASc score.

## Abbreviations

AUC: area under the curve
BNP: B-type natriuretic peptide
CI: 95 % confidence intervals
LAA: left atrial appendage
LVEF: left ventricular ejection fraction
NVAF: nonvalvular atrial fibrillation
OR: odds-ratio
ROC: receiver operating characteristic
SEC: spontaneous echo contrast
